# Report of Surgical Cases

**Published:** 1846-04-01

**Authors:** F. H. Hamilton

**Affiliations:** Professor of Surgery in Geneva Medical College


### Report of Surgical Cases.

By F. H. HAMILTON, M. D., Professor of Surgery in Geneva Medical College.

Dr. Flint—Dear Sir:—I am happy, in compliance with your request, to report to you something from our surgical dispensary connected with Geneva College. Like college dispensaries elsewhere, the cases presented, and operations made are generally of a minor character, and yet, perhaps to the student none the less useful. During the eight weeks of my term, 107 cases have been presented, upon the large majority of whom, operations have been made.

Among which, were three cases of Labium Leporinum, *in all of which I used the common interrupted sutures*, adhesive plasters, &c., as by me recommended in a former number of your Journal—and with complete success. The first was a child 6 months old, daughter of D. Yakely, Fayette, Seneca Co. The cleft in the upper lip was wide, and corresponded with a similar cleft through the whole depth of the palatine bones and soft palate. Has never nursed—healthy.

*Operation*, *Jan.* 10, 1846. During the operation the child lost about ii℥ of blood and became pale, but soon rallied. I added to my usual dressings a *fine suture* quite upon the lip, to make the coaptation more perfect. On the 14th I removed all the sutures—union complete—the *fine suture* has aided materially in the perfection of the operation. From the second day until the tenth I renewed the adhesive straps daily—afterwards every other day for a week.

Mrs. Frantz, aged about 35—congenital Labium Leporinum; fissure only about 5 lines deep. In this case I resorted to a modification of the operation, which I flatter myself may prove a valuable suggestion in other similar cases. With a narrow sharp pointed bistoury I transfixed the lip three lines above the upper angle of the fissure, carrying the knife from this point obliquely downward and outward to within three lines of the margin of the lip; having thus made a straight slit parallel to one side of the fissure. I now introduced the knife again at the top of the incision and carried it parallel to the other side of the fissure, in the same manner as before. The triangular border thus intercepted immediately fell to the level of the natural lip, leaving above a triangular hole; to close which, a strong suture was passed through the lip on each side of the base of the triangular opening and drawn snugly together. The portion thrown down, being now inclined to fall too far, it was slightly supported by a single fine suture. One adhesive plaster completed the dressings.

The result is a complete obliteration of the fissure, *leaving nothing of that slight depression which almost invariably remains from a successful operation.*

C. D. Palmyra, aged 24—congenital fissure; does not extend through bones.

*Operation*, *Jan.* 30, 1846. Instructed by the favorable result of the case last mentioned, I determined here, also, if possible, to prevent the persistence of the usual unseemly depression. But the fissure was too deep to allow me to proceed in precisely the same manner. I therefore scarified the edges of the fissure in the usual way: then I seized upon the lip and introducing the knife about four lines above the junction of the fissure with the straight border of the lip, carried it horizontally outward and parallel with the straight border of the lip, 4 lines. The same was repeated upon the opposite margin of the fissure. The ends of these two flaps were now squared and brought together by a suture, leaving the lower margin perfectly straight. The balance of the operation was completed according to my usual mode, with sutures, adhesive plasters, &c.

The result of this case is most gratifying, and determines me to adopt this modification in all similar cases hereafter.

*Hereditary Elephantiasis Tuberculosa.* This is the only instance of this terrible malady it has been my fortune to meet with on this continent, and north of the 35th parallel.

Mrs. J. S., aged 30. Born and resided in Putnam Co. in this State until the last 16 years: since then has resided in Wayne Co.: has never lived South. A brother has two similar tumors on head. The father, now 60 years old, has a number; and the grandmother on the fathers side had the same. All are natives of a northern climate. With Mrs. S. the tumors commenced on the upper part of face, about 12 years since, and have gradually multiplied. They now vary in size from a small shot to a Madeira nut, being larger on the crown and gradually diminishing in size down the face, covering completely with scarce the interval of one line the top and sides of the head, forehead, &c. They are of a pale pink color and possess the feel and form of pretty firm adipose tumors; painless and soreless unless injured. One has ulcerated spontaneously and disappeared—one she removed by ligation. Her complexion is fair—hair light—general appearance delicate, but healthy. She is well, except that she has occasionally an acid stomach.

I considered a cure impossible, but with a view of retardation, directed a solution of nit. arg. gr.i, water iii℥, as a daily lotion to the tumors, and antacids internally.

*Ptosis. Dec. 20th.* Miss E. Merrithew, aged 16—complete Ptosis in both eyes as a result of chronic inflammation. An operation has once been made, but too little was removed and no benefit was obtained. I removed from each upper lid a piece of integument of the length of the lid, elliptical, and half an inch wide in the center. Secured lips of wound with four sutures on each lid. Result, complete success.

*Ptosis.* Mrs. ——— of ———, aged 45. Result of chronic opthalmia—partial—conjunctiva thickened. Removed a piece of integument of the same length but not as wide; used three sutures. Result, successful.

*Pterygeum.* H. P., South Sodus—aged 26. Commenced at inner canthus three years since; is now broad and fleshy; its apex encroaches two lines upon the cornea.

I advised him of my usual want of success in these cases, whether iterfered with by astringents, caustics or the knife, but he urged the operation. With bull-dog forceps and scissors I excised it from the margin of the cornea inward four lines, and in breadth exceeding by one line the margin of the pterygeum, determining to give the case a fair trial.

In six weeks the patient was before the class and the pterygeum was as large as before the operation. He now consented to let it alone; probably it will never materially impair vision. I have occasionally succeeded in effectually eradicating pterygea, yet *generally* my interference has rendered them worse. I prefer the scissors to caustic.

Several cases of *cataract* upon which I operated by *laceration*, possess no special interest.

Of *Aneurism* by *Anastamosis* 5 cases were removed either by ligature or knife; the ages of the patients ranging between five months and three years. A very interesting case of varicose tumor on the arm of Mr. P. of Seneca Falls, I shall report as soon as the result is known.

*Necrosis of shaft of Humerus.* Henry King, aged 15. Disease of 7 years standing. With Heys-saw, a chisel and mallet, a longitudinal opening was made in the new shaft of four inches in length, and a sequestrum of about 5 inches removed. The wound has healed kindly.

